# Supplementary material for: Angelica sinensis polysaccharide as potential protectants against recurrent spontaneous abortion: focus on autophagy regulation
Source: Front Med (Lausanne). 2025 Jan 15;12:1522503. doi: 10.3389/fmed.2025.1522503 (PMC11774876; doi:10.3389/fmed.2025.1522503)
Supplement: Supplementary material S1 — The main instruments used during the LC-MS process, along with their models/specifications and manufacturers. [file Supplementary_file_1.zip › Metabolomics sequencing data FC1.2/8.代谢通路分析/Exposure_Ctrl/all/KEGG/mmu00260.html]

KEGG PATHWAY: Glycine, serine and threonine metabolism - Mus musculus (house mouse)


# Glycine, serine and threonine metabolism - Mus musculus (house mouse)


[
Pathway menu
| Organism menu
| Pathway entry
| Show description
| Download
| Help
]

Serine is derived from 3-phospho-D-glycerate, an intermediate of glycolysis [MD:M00020], and glycine is derived from serine. Threonine is an essential amino acid, which animals cannot synthesize. In bacteria and plants, threonine is derived from aspartate [MD:M00018].


##### Option

Scale:


100%

Image resolution:


 High

##### Search

##### ID search

##### Color

##### Module

Complete only
Including 1 block missing
Including any incomplete

- Pathway modules
  - Amino acid metabolism
    - Serine and threonine metabolism
      - M00020
        Serine biosynthesis
      - M00018
        Threonine biosynthesis
      - M00555
        Betaine biosynthesis
      - M00033
        Ectoine biosynthesis
      - M00919
        Ectoine degradation
    - Cysteine and methionine metabolism
      - M00338
        Cysteine biosynthesis


KGML

Image (png) file 1x
